# Nutritional Programming of the Lifespan of Male Drosophila by Activating FOXO on Larval Low-Nutrient Diet

**DOI:** 10.3390/nu15081840

**Published:** 2023-04-11

**Authors:** Yue Gao, Xingyi Cheng, Yao Tian, Zhixiao Yuan, Xiaolan Fan, Deying Yang, Mingyao Yang

**Affiliations:** 1 Institute of Animal Genetics and Breeding, Sichuan Agricultural University, Chengdu 611130, China; drgaoyue@163.com (Y.G.); chengxingyisicau@163.com (X.C.); tianyao0210@163.com (Y.T.); yzx15699336321@163.com (Z.Y.); xiaolanfan@sicau.edu.cn (X.F.); dnaydy@126.com (D.Y.); 2 Farm Animal Genetic Resources Exploration and Innovation Key Laboratory of Sichuan Province, Sichuan Agricultural University, Chengdu 611130, China

**Keywords:** low-yeast diet, nutritional programming, male, *Drosophila*, lifespan, dFOXO

## Abstract

Nutrition during the developmental stages has long-term effects on adult physiology, disease and lifespan, and is termed nutritional programming. However, the underlying molecular mechanisms of nutritional programming are not yet well understood. In this study, we showed that developmental diets could regulate the lifespan of adult *Drosophila* in a way that interacts with various adult diets during development and adulthood. Importantly, we demonstrated that a developmental low-yeast diet (0.2SY) extended both the health span and lifespan of male flies under nutrient-replete conditions in adulthood through nutritional programming. Males with a low-yeast diets during developmental stages had a better resistance to starvation and lessened decline of climbing ability with age in adulthood. Critically, we revealed that the activity of the *Drosophila* transcription factor FOXO (dFOXO) was upregulated in adult males under developmental low-nutrient conditions. The knockdown of dFOXO, with both ubiquitous and fat-body-specific patterns, can completely abolish the lifespan-extending effect from the larval low-yeast diet. Ultimately, we identify that the developmental diet achieved the nutritional programming of the lifespan of adult males by modulating the activity of dFOXO in *Drosophila*. Together, these results provide molecular evidence that the nutrition in the early life of animals could program the health of their later life and their longevity.

## 1. Introduction

Nutrition plays an important role in shaping metabolic health and aging [1,2]. Dietary restriction (DR) without malnutrition is the most classic and reproducible means of delaying aging and prolonging lifespan [2,3]. At present, numerous dietary intervention schemes have been reported according to different nutritional components and different stages of nutritional intervention [2,4]. Several reports have shown that dietary intervention in early adulthood strongly shapes metabolism, fecundity, and longevity in later life, and this early life nutritional memory is often irreversible [5,6,7]. However, most studies of dietary interventions have focused on adulthood, and only a few focus on diets during developmental stages. There are many reports revealing that the early-life (developmental) environment can exert permanent and powerful effects in shaping adult phenotypes [8,9]. In fact, long-term epidemiological studies have revealed that a suboptimal nutritional environment during developmental stages (pregnancy or infancy) is associated with a higher risk of type 2 diabetes (T2D) and cardiovascular disease (CVD) mortality. This long-term effect of nutrition is termed nutritional programming [10,11,12]; the nutritional stimuli cause long-term or lifelong structural and functional changes in organisms, including body size, physiological state, disease risk, behavior and lifespan. Several pieces of experimental evidence also revealed that developmental nutrition can modify lifespan and adult health. For example, a maternal low-protein diet during suckling in male mice protects offspring against the life-shortening effect of an obesity-inducing diet and increases longevity, presumably via effects on milk quality [13]. In *Drosophila*, depriving third-instar larvae of yeast, which is the main dietary source of protein, produces smaller-sized, less fecund adults without significantly altering lifespan [14]. Developmental nutrition affects virgin lifespan, but not that of mated flies, and alters reproductive investment patterns in adults, which depend on the adult environment [15]. In addition, the dilution of a dietary yeast extract throughout larval development extends adult lifespan in *Drosophila* [16]. Therefore, nutrition during critical or sensitive periods of development may “program” the organism’s lifelong physiological health and lifespan. However, the underlying molecular mechanisms of early nutritional programming on adult health and longevity are largely unknown.

It is generally believed that the underlying mechanisms of nutritional programming include permanent structural changes in organs by early life nutrition, such as shaping the cell number, size, and structure of islets [17,18], the kidney [19], and the heart [20]. There is also evidence that supports cellular senescence caused by oxidative stress and macromolecular damage [21,22,23]. Epigenetic changes that program gene expression in later life are important for nutritional programming [24,25,26,27]. Restricting dietary yeast extract during development can extend the lifespan of the fruit fly itself and its conspecific neighbors by reducing the production of lipid autotoxins [16]. However, the existing results do not fully explain the effects of nutritional programming on health and lifespan. In particular, there is a lack of studies exploring the mechanisms of nutritional physiology and aging. Therefore, we investigate the molecular network of the nutritional regulation of aging from the perspective of the long-term effects of developmental nutrition.

The evaluation of the effects of anti-aging interventions, such as DR, should not only consider the extension of lifespan (maximum lifespan) but should also focus on the improvement of physiological function, that is, health span. Physiological functions, such as motor/physical function, body composition, glucose tolerance/insulin sensitivity, and inflammation/oxidative stress, can be utilized to assess the biological effects of aging and the effectiveness of treatments [28]. Glucose, insulin, and insulin-like growth factor 1 (IGF-1) levels are often used as indicators of glucose metabolism [29,30]. Studies have shown that DR reduces fasting plasma insulin levels, IGF-1 levels, and blood glucose levels in mice [29,30,31]. Interestingly, male mice retained a glucose metabolic memory of DR feeding in early adult life in terms of improved glucose tolerance [29,30]. This improved glucose tolerance persisted even at 10 months, when there were no differences in fasting insulin levels and insulin sensitivity [30].

Nutrient-sensing pathways are essential parts for organisms to respond to environmental nutrient fluctuations and adjust their metabolism in time to maintain energy homeostasis [32]. These pathways, including the mechanistic target of rapamycin (mTOR), insulin/IGF-1-like signaling, and sirtuins mediating the lifespan-extending effects of DR, have been identified in multiple model organisms [33]. The modulation of key components in nutrient-sensing signaling pathways—such as insulin-like receptor (InR) [34] and chico [35] of IIS, TSC1, TSC2, ribosomal protein S6 kinase (S6K) [36], and 4EBP [37] of mTOR pathway and Sirt6 [38]—has been shown to regulate the lifespan of *Drosophila*. In addition, Forkhead box O (FOXO) transcription factors are master regulators that translate insulin, growth factor, and nutrient and oxidative stress signals into downstream-specific gene expression programs [39]. Signals from multiple signaling pathways involved in aging and longevity are integrated by DAF-16/FOXO, including the insulin/IGF-1 signaling pathway, TOR signaling pathway, AMPK pathway, JNK pathway, and germline signaling pathway [40]. It was reported that FOXO3 mediated the lifespan-extending effect of dietary restriction in mice [41] and was also found to be associated with extended human lifespan [39]. *Drosophila* has only one FOXO gene (dFOXO), which is homologous to mammalian FOXO3a. When cells are stimulated by insulin, dFOXO is phosphorylated by AKT, leading to cytoplasmic retention and the inhibition of its transcriptional activity [42]. Previous studies have shown that FOXO is required for programming the lifespan-shortening effects of a high-sugar diet in early life [7]. Together, FOXO are important targets for regulating healthy aging and longevity.

*Drosophila* is an ideal model for investigating developmental nutrition and aging as its developmental stages are undisturbed by the maternal environment. In this paper, we systematically explore the role of developmental diets in shaping *Drosophila* lifespan via different die combinations during development and adulthood. We found that a developmental diet regulates the longevity of adult *Drosophila* in a way that interacts with the adult diet. Specifically, a low-yeast diet during larval stages was able to extend the health span and lifespan of male fruit flies under nutrient-replete conditions in adulthood through nutritional programming. Importantly, we uncovered that a low-yeast diet during developmental stages stimulates the activity of *Drosophila* transcription factor FOXO in adults, which is required for the lifespan-extending effect in flies. Our results reveal a novel molecular mechanism of dFOXO for the nutritional programming of the lifespan in male *Drosophila*.

## 2. Materials and Methods

### 2.1. Fly Stock and Husbandry

The wild-type *Dahomey*, *W^Dah^,* and *W^1118^* stocks were gifts from Piper and Partridge’s lab [43]. Akt mutant (CG4006, BL11627), S6K mutant (CG10539, BL11713), Thor mutant (CG8846, BL9559), and dFOXO-RNAi (CG3143, BL32427) were obtained from the Bloomington Drosophila Stock Center (https://bdsc.indiana.edu, accessed on 6 September 2021). The mutant lines of Akt, S6K, and Thor were backcrossed into *W^1118^* for at least two generations. The dFOXO RNAi line was backcrossed into *W^1118^* for six generations. Ppl-GAL4 was a gift from the Xun Huang lab and was backcrossed into *W^1118^* for six generations. Both Chico mutant [44] and dFOXO mutant [43,45] were gifts from Partridge’s lab and were backcrossed into the control *W^Dah^* stock for at least six generations. *W^Dah^* stock was derived by backcrossing *W^1118^* into the outbred wild-type *Dahomey* background. For the experiment of knocking down dFOXO in the fat body, the maternal ppl-GAL4 flies were crossed with the paternal dFOXO-RNAi flies, and the F1 generation was used. The corresponding control flies were the offspring of the cross between ppl-GAL4 and *W^1118^*. All stocks were maintained at 25 °C on a constant light:dark cycle of 12:12 h at 65% humidity. In the experiments, SY foods were used [46]. Different yeast concentrations of 0.05SY, 0.2SY, 1SY, and 3SY contained 10 g/L agar; 50 g/L sucrose; and 5 g/L (0.05SY, very low yeast), 20 g/L (0.2SY, low yeast), 100 g/L (1SY, normal yeast), or 300 g/L yeast (3 SY, high yeast) (Yeast brand: ANGEL YA100); 30 mL/L nipagin; and 3 mL/L propionic acid. For the *holidic* medium of 100N50S Yaa (with amino acids in the ratio of Yaa of 100 mM biologically available nitrogen), referring to Piper et al. [47]. For all experiments, flies were reared at a density of 40 µL egg sedimentation solution per bottle on a 0.2SY, 1SY, or 3SY medium. The 1SY medium was used as the control diet in all experiments. Enclosed adults were collected over a 12 h period. Flies were mated for 48 h on a 1SY medium before being sorted into separate sexes. Adult flies were cultured on a 0.05SY, 0.2SY, 1SY, 3SY, or 100N50S Yaa medium depending on the experiment.

### 2.2. Lifespan Analysis

**Figure 1:**
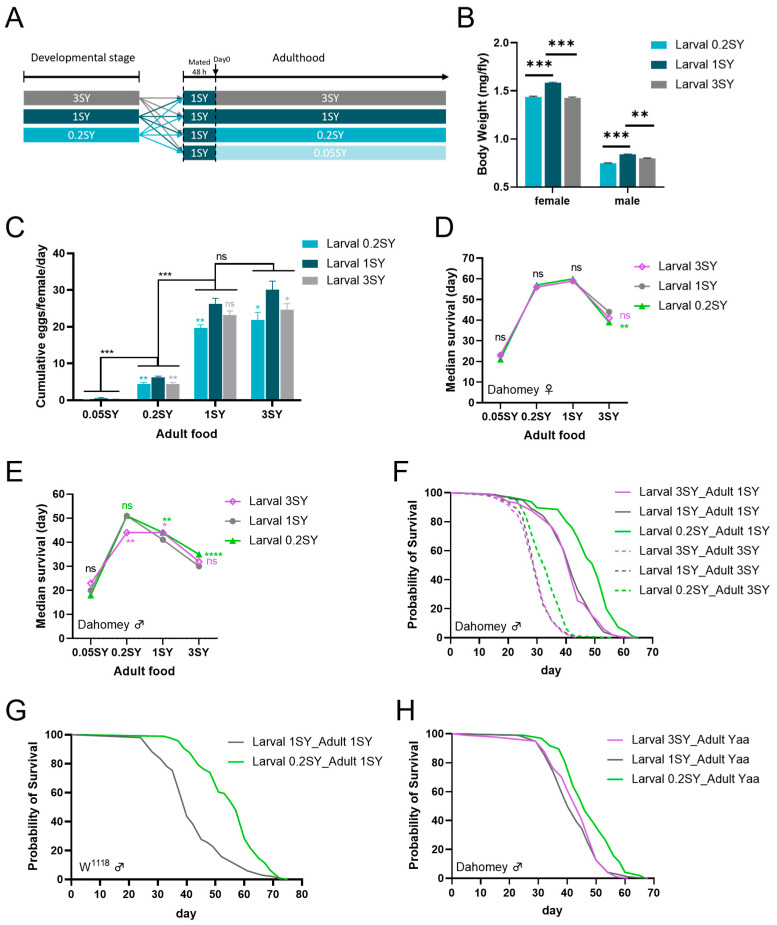
Developmental dietary regulates adult *Drosophila* lifespan and physiology. (**A**) Diet schemes used in this study. (**B**) Body weight of adult males after mating for 48 h (day 0) under different developmental diets of 0.2SY, 1SY, or 3SY. (**C**) Egg production of female flies at day 7 under different yeast concentrations during development and adulthood. (**D**) Median lifespans of females on different yeast concentrations during development and adulthood. (**E**) Median lifespans of males on different yeast concentrations during development and adulthood. (**F**) Low-yeast diets (0.2SY) during development significantly extend the lifespan of *Dahomey* male flies. (**G**) Low-yeast diets (0.2SY) during development significantly extend the lifespan of *W^1118^* male flies. (**H**) *Dahomey* males with low-yeast diets (0.2SY) during development had a longer lifespan on the *holidic* medium of 100N50S Yaa in adulthood. All results shown, except where indicated, are of *Dahomey* flies. Body weight in (**B**) and egg production in (**C**) shown as mean ± SEM. *p*-values were determined using an unpaired Student’s *t*-test (for body weight) or a one-way ANOVA, followed by a Tukey’s multiple comparison test (for egg production data). Lifespan differences were assessed using the log-rank test. Lifespan data can be seen in the Tables S1–S4 and S7. * *p* < 0.05; ** *p* < 0.01; *** *p* < 0.001; **** *p* < 0.0001; *ns*: not significant (*p* > 0.05).

Female or male flies were randomly allocated to the experimental food treatments and housed in plastic vials containing food at a density of 10 flies per vial, with 10 vials per condition (*n* = 100). Flies were transferred to a fresh food source every 2–3 days, during which any deaths and censors were recorded. The lifespan experiments were performed three times independently (Figures 1F and 6C), with each experiment containing 100 flies. The rest of lifespan experiments were completed two times independently. The log-rank test was used for the comparison of survivorship data (see Tables S1–S9).

### 2.3. Body Weight Assay

Flies were collected with CO_2_. The body weight was measured in groups of ten flies, with at least six replications. The total number was over 60 flies per condition. A Student’s *t*-test was used for the comparison of differences.

### 2.4. Food Intake Assay

Food intake was measured using the excreta quantification (EX-Q) method developed by our lab [48]. Flies were cultured on the adult medium for 10 days and then transferred to the dye-labeled food with 1% Erioglaucine disodium salt (Sigma Aldrich, 861146, Shanghai, China) in food intake vials and kept for 24 h. The excreta were collected and quantified by measuring absorbance at a wavelength of 630 nm.

### 2.5. Fecundity Assay

The number of eggs laid over 24 h periods (days 7–8) was counted. For each condition, 10 vials were counted. Each vial contained 10 female flies. The egg-laying differences were assessed using a one-way ANOVA followed by a Tukey’s multiple comparison test.

### 2.6. Climbing Ability Assay

About 100 flies were prepared for each condition and measured once a week from the first week until the fourth week. The determination of the climbing ability was conducted according to Sofola et al. [49]. Fruit flies were shaken to the bottom of the measuring tube. Each condition was performed simultaneously. The flies were allowed to climb vertically upwards for 40 s, and then pictures were taken. The bottom was recorded, as were the top and total number of fruit flies in the measuring tube. A performance index (PI) defined as ½ (*N_tot_* + *N_top_* − *N_bottom_*)/*N_tot_*) was calculated. Five repeat measurements were performed for each condition.

### 2.7. Starvation Assay

Male flies mated 48 h after eclosion were transferred to plastic vials containing the starvation medium (10 g/L agar, 30 mL/L nipagin and 3 mL/L propionic acid) of 10 flies per vial, with 10 vials per condition. The number of deaths was recorded twice a day without transferring to the fresh medium. A log-rank test was used for the comparison of survivorship data.

### 2.8. RNA Isolation and RT–qPCR

Total RNA was extracted from 10 flies using TransZol (TransGen Biotech, ET101-01, Beijing, China) according to the manufacturers’ instructions. cDNA was synthesized (TransGen Biotech, AW311-02, Beijing, China), and the real-time quantitative PCR experiment was performed (TransGen Biotech, AQ711-01, Beijing, China) according to the manufacturers’ instructions. The relative expression of the target gene was calculated using 2^−ΔΔCt^, and each independent sample was repeated three times. rp49 was used as an internal reference gene. The primers are listed in Table 1.

### 2.9. Western Blot

Total protein was extracted from 15 fruit flies with the Tissue or Cell Total Protein Extraction Kit (Sangon Biotech, C510003, Shanghai, China). Proteins were separated on SDS polyacrylamide gels and blotted onto polyvinylidene fluoride membrane. Membranes were blocked in 5% milk/TBST for 2 h at room temperature, and primary antibodies were incubated overnight at 4 °C. The used primary antibodies were as follows: α-tubulin antibody (Immunoway, YM3035, 1:3000, Suzhou, China,), pAKT (Immunoway, YP0490, 1:5000, Suzhou, China), AKT (Immunoway, YM3618, 1:5000, Suzhou, China), anti-pS6K (CST, 9209S, 1:1000, Shanghai, China), anti-S6K (Immunoway, YT3555, 1:1000, Suzhou, China), anti-pFOXO (Immunoway, YP0113, 1:600, Suzhou, China), and rabbit anti-FOXO (made by our lab, 1:1000). According to different primary antibodies, membranes were incubated with goat anti-mouse (Proteintech, SA00001-1, 1:6000, Wuhan, China) or goat anti-rabbit (Proteintech SA00001-2, 1:8000, Wuhan, China) for 1 h. Membranes were washed with TBST before and after each antibody incubation. The gray value was measured using ImageJ v1.53k software.

### 2.10. Carbohydrate and Triglyceride Assay

At least 6 replicates were collected for each condition. Each replicate contained 10 flies. The average body weight of the fruit flies was measured. For the triglyceride content quantification, the samples were homogenized in 500 µL alcohol per EP tube. They were centrifuged at 12,000 rpm 4 °C for 10 min, and the supernatant was removed for measurements. Then, the triglyceride content quantification was performed following the protocol of the triglyceride assay kit (Nanjing Jiancheng Bioengineering Institute, Nanjing, China). The triglycerides per body weight value was calculated. For the carbohydrate quantification, the samples were homogenized in a 200 µL PBST (0.2% Triton X-100/PBS, Triton X-100: Solarbio, T8200, Beijing, China. PBS: Solarbio, P1000, Beijing, China) solution per tube. They were heated at 70 °C for 5 min to inactivate the enzyme in a mixed liquid. They were centrifuged at 12,000 rpm 4 °C for 10 min, and the supernatant was removed for measurements. Then, whole-fly glucose quantification was performed following the protocol of the D-Glucose Assay Kit (Megazyme, K-GLUC (GOPOD-FORMAT), Wicklow, Ireland). For the whole-fly glycogen determination, the supernatant of the samples was incubated with amyloglucosidase (Sigma Aldrich, A7420, Shanghai, China) at 37 °C for 10 min. The total glucose was measured, and the basal glucose content was subtracted to obtain the glycogen content. Finally, glucose per body weight and glycogen per body weight were calculated.

### 2.11. Oil Red O Staining of Gut Fat

For each condition, 15–20 *Drosophila* guts were dissected in PBS and fixed in 4% paraformaldehyde/PBS for 30 min. After being rinsed twice with distilled water, the guts were stained with 5% Oil Red O/isopropanol (Oil Red O/isopropanol solution was pre-filtered with a 0.45 µm syringe filter) for 30 min. The stained guts were rinsed twice with distilled water and mounted in glycerol. The entire gut and the anterior midgut were photographed under a microscope. The gray value of the anterior midgut was calculated using ImageJ v1.53k software.

## 3. Results

### 3.1. Developmental Diet Interacts with Adult Diet to Regulate Lifespan and Physiology in Adult Drosophila

We explored the effects of developmental nutrition on adult lifespan and physiological phenotypes in *Drosophila*. As yeast is an important source of proteins, vitamins, minerals, and sterols in the *Drosophila* diet [50,51], wild-type *Dahomey* flies were cultured on sugar–yeast food with changes in yeast concentration during the developmental stages. The sugar–yeast foods during the developmental stages were divided into three groups, which were: the low-yeast group, 0.2SY (20 g/L yeast); the normal-yeast group, 1SY (100 g/L yeast); and the high-yeast group, 3SY (300 g/L yeast). After eclosion, they were fully mated on a normal-yeast medium for 48 h. Then, the flies of all conditions were transferred to adult diets of four yeast concentrations of 0.05SY (very-low-yeast, 5 g/L yeast), 0.2SY, 1SY, and 3SY (Figure 1A). The results show that the larvae developed on the 1SY diet achieved their maximum body weight, while the larvae developed on yeast concentrations below or above that of 1SY had a significantly reduced adult body weight at day 0 of adulthood (Figure 1B and Figure S1A). Similar results were also observed in female fecundity. Flies whose larvae were raised on a 1SY diet displayed the best fecundity among the three adult diets, while flies whose larvae were developed with concentrations below or above that of 1SY had significantly reduced female egg production (Figure 1C). This is different from the typical egg production phenomenon, which is boosted with the increase in yeast concentrations of the adult diet (Figure 1C). These results are consistent with previous findings [16,52,53].

We then determined the longevity of the resulting adult *Drosophila*. The results show that developmental diets could regulate the lifespan of adult *Drosophila* in a way that interacts with adult diets. Specifically, the lifespan of female flies fed with low yeast (0.2SY) during the developmental stages had no significant difference with that of flies on the adult diet of 1SY, while the lifespan was significantly shortened on the adult diet of 3SY compared to that achieved on normal yeast (1SY) during development (Figure 1D and Figure S1B and Table S1). Interestingly, we found that male flies with low-yeast diets (0.2SY) during developmental stages lived longer under nutrient-replete conditions (1SY and 3SY) in adulthood (Figure 1E,F and Figure S1B and Tables S2 and S3). We termed 0.2SY of “the low-yeast diet during developmental stages” as 0.2SY LYDDS.

To confirm the above outcome, a different wild-type fly, *W^1118^*, was used. The results indicate that male flies with 0.2SY LYDDS had a longer lifespan under normal food conditions in adulthood (Figure 1G and Table S7). In addition, we used 100N50S of the pure chemical substance, a *holidic* medium [47], in adulthood, which is similar to the yeast–amino acid ratio in SY food. Compared with to fed with normal yeast (1SY) and high yeast (3SY) during their development, *Dahomey* male flies with 0.2SY LYDDS displayed a longer lifespan in a nutrient-replete adult *holidic* diet (Figure 1H and Table S4).

### 3.2. Overlap between Nutritional Programming and Adult DR in Lifespan Extensions

**Figure 2:**
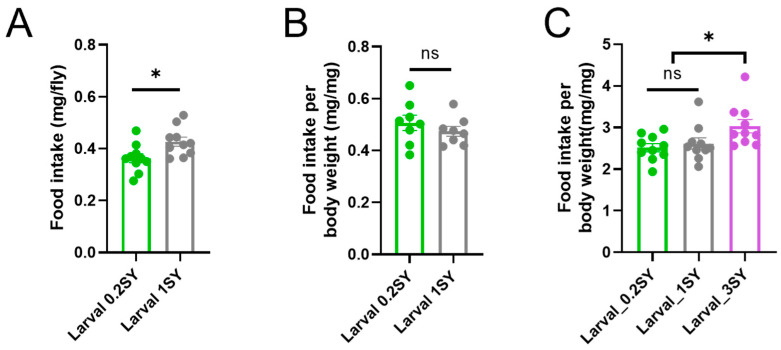
Low-yeast diets during developmental stages do not affect the food intake per bodyweight of adult male *Drosophila*. (**A**,**B**) Food intake at day 10 for male flies which were raised on a 1SY or 0.2SY diet during their developmental stages of 1SY food within 24 h. (**A**) Total food intake. (**B**) Food intake per body weight. (**C**) Food intake per body weight of male flies fed with different yeast concentrations during developmental stages cultured on Yaa food for 10 days as adults. All results shown are of WT *Dahomey* male flies. All data are shown as mean ± SEM. Each point represents 1 vial of 10 flies. *p*-values were determined by an unpaired Student’s *t*-test. * *p* < 0.05; *ns*: not significant (*p* > 0.05).

We also investigated whether the lifespan-extending effect of male flies fed a larval low yeast diet acted as a DR mimetic. Given the strong correlation between lifespan and food intake, we first measured the food intake of adult male flies. The results show that male flies had a significantly lower total food intake when comparing low-yeast (0.2SY) and normal-yeast (1SY) diets during development (Figure 2A). However, there was no significant difference in food intake per body weight between them (Figure 2B,C). When further analyzing the median lifespan, we found that male flies reached a maximum median lifespan on an adult diet of 0.2SY. That is, 0.2SY had the optimal dietary yeast for DR of male *Drosophila* in adulthood, while 0.2SY LYDDS did not further extend the maximum median lifespan achieved in adulthood under optimal dietary conditions (Figure 1C and Figure S1B). This suggests that the mechanisms underlying the lifespan extension of developmental low nutrition partially overlap with DR in adulthood.

### 3.3. Low Nutrition during Development Promotes the Health Span of Male Drosophila

**Figure 3:**
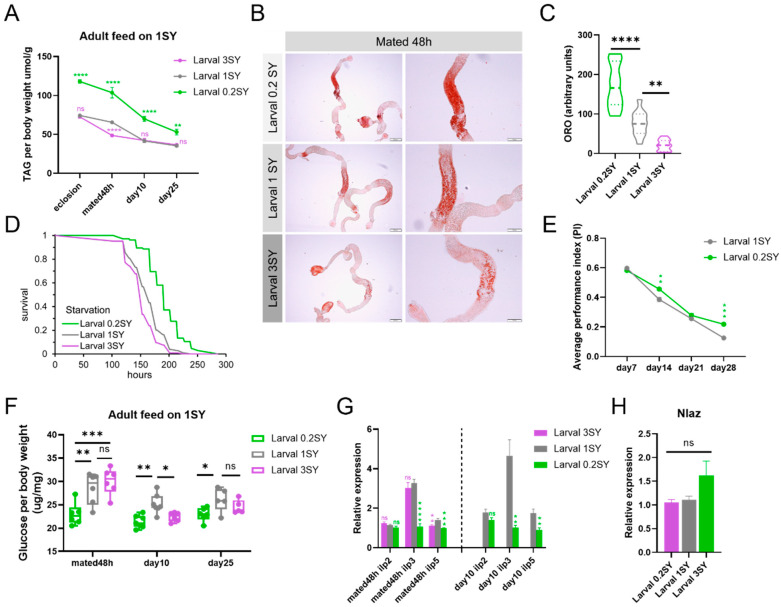
Developmental low-yeast diets promote the health of male *Drosophila*. (**A**) Total TAG content per body weight of male flies at different larval yeast concentrations. Adults were cultured on normal yeast (1SY diet). Samples were collected at different time points. (**B**) Oil Red O (ORO) staining of the gut lipids of male flies raised on 0.2SY, 1SY, or 3SY diets during their developmental stages. Pictures in the left column are whole gut tissue. Scale bar = 200 µm. Pictures in the right column are anterior midgut. Scale bar = 100 µm. (**C**) Quantification of the ORO signal in the anterior midgut. Flies raised on the 0.2SY diet had significantly higher gut lipid storage compared to flies raised on the 1SY and 3SY diets during development stages. At least 7 guts were quantified for each condition. (**D**) Starvation assay of male flies under different developmental nutritional conditions after mating for 48 h. Male flies developed on the 0.2SY diet were significantly more resistant to starvation compared to flies developed on the 1SY and 3SY diets. (**E**) Climbing ability decline with the age of male flies was significantly delayed under 0.2SY LYDDS. (**F**) The glucose levels of male flies of different ages raised on different yeast concentration diets during development. (**G**) The transcriptional level of insulin-like peptides in male flies of different ages raised on different yeast concentration diets during development. (**H**) Transcriptional level of insulin residence marker gene Nlaz of male flies under different yeast concentration conditions during development after mating for 48 h. All results shown are of WT *Dahomey* male flies, and the adult diet is 1SY. All data except in (**B**–**D**) are shown as mean ± SEM. Each point of (**F**) represents 1 vial of 10 flies. *p*-values were determined by an unpaired Student’s *t*-test (for a, (**E**–**H**)) or a one-way ANOVA, followed by a Sidak multiple comparisons test (for quantification data of ORO signal). The differences in anti-starvation survival data were assessed using the log-rank test. * *p* < 0.05; ** *p* < 0.01; *** *p* < 0.001; **** *p* < 0.0001; *ns*: not significant (*p* > 0.05).

To explore the effects of a low-yeast diet during the developmental stages on the physiological health of male flies, we investigated the metabolic status by assessing their energy storage. The results show that compared to the control groups, adult males fed with 0.2SY LYDDS had significantly more lipid storage, as indicated by the significantly higher whole-fly triacylglyceride (TAG) levels per body weight after eclosion (Figure 3A). Lipid accumulation in the anterior midgut is sensitive to dietary nutrition, and DR increased the lipid accumulation in the anterior midgut, which is good for animal health span [54]. Therefore, we stained the gut with Oil Red O (ORO) to directly visualize the TAG content. The results demonstrate that there was a significant increase in lipids stored in the anterior midgut of males fed with 0.2SY LYDDS after mating for 48 h after eclosion (Figure 3B,C). Consistent with these results, male flies fed with 0.2SY LYDDS were more resistant to starvation compared to the flies fed with 1SY and 3SY diets during their development (Figure 3D). We further determined how fat deposition changes with age in different developmental yeast conditions. The lipid deposition of all conditions in the whole fly and anterior midgut of males decreased with age. However, the difference between low yeast (0.2SY) and other yeast concentrations (1SY and 3SY) during developmental stages diminished with age, regardless of whether they were fed with the 1SY or 3SY diets (Figure 3B,C and Figure S2A,C,D). Taken together, these results suggest that males fed with 0.2SY LYDDS store more energy in early adulthood. Regarding the improvement of other physiological functions in male flies fed with 0.2SY LYDDS, climbing ability decline with age was significantly delayed (Figure 3E).

Previous studies have shown that undernutrition during development increases the risk of metabolic syndrome and disease, including T2D and CVD in adults [12]. However, lower glucose levels and insulin levels are the typical phenotypes of DR-extending lifespan [3,29,30,31]. To verify whether a developmental low-yeast diet can cause abnormal glucose metabolism in *Drosophila*, we measured the glucose and glycogen levels in adult flies. The results show that there was no significant difference in glycogen levels (Figure S2B); however, the glucose levels were significantly lower in male flies fed with 0.2SY LYDDS compared to those fed with normal diets during development (Figure 3F). Furthermore, we examined the transcriptional levels of insulin-like peptides ilp2, ilp3, and ilp5, which are considered the main regulators of sugar homeostasis. Consistent with a lower overall glucose level, we found that the expression of ilp3 and ilp5 was significantly lower in males fed with 0.2SY LYDDS after mating for 48 h after eclosion and at 10 days (Figure 3G).

Lipocalin Neural Lazarillo (NLaz) is considered as an insulin residence marker in *Drosophila* [55,56] and is also involved in modulating the IIS pathway and regulating longevity and stress resistance in *Drosophila* [57]. We found that the transcriptional level of NLaz was unchanged in male flies compared with those fed with low-yeast and other yeast concentrations during their developmental stages (Figure 3H). These results suggest that 0.2SY LYDDS does not cause insulin resistance in adult *Drosophila*. Therefore, 0.2SY LYDDS provides lifelong health benefits to male *Drosophila*.

### 3.4. The Lifespan-Extending Effect of a Low-Yeast Developmental Diet Is Partially Dependent on the IIS and mTOR Signaling Pathways

**Figure 4:**
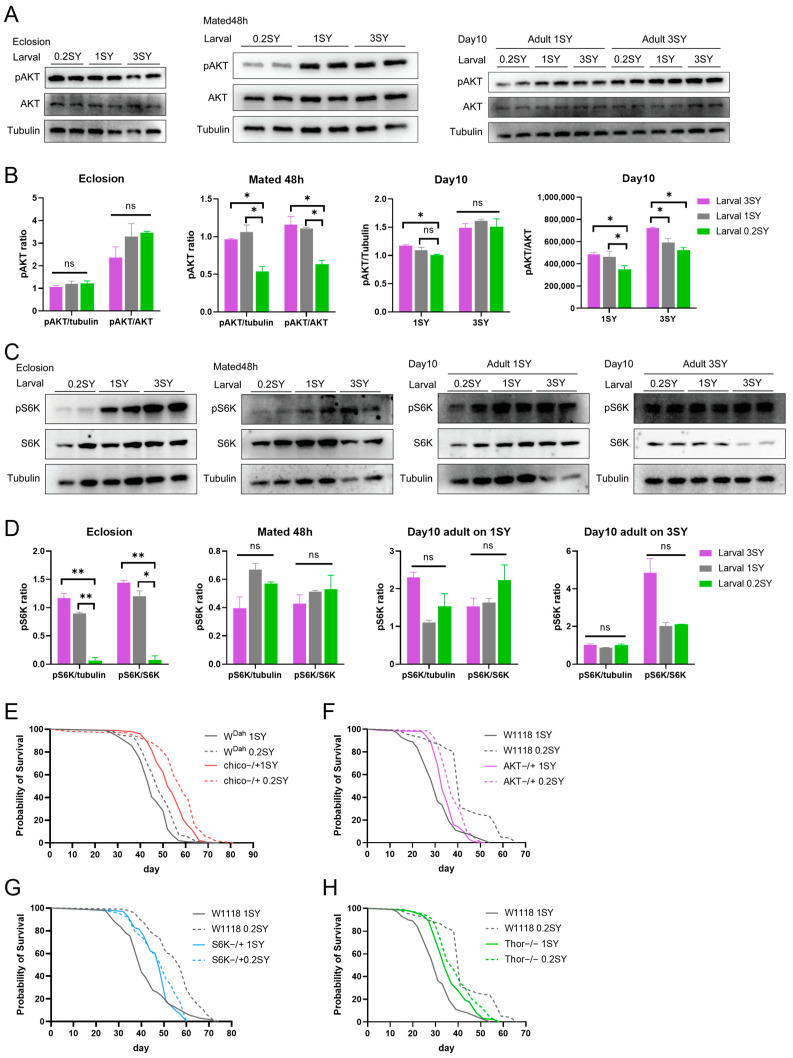
Developmental low-yeast diets inhibit nutrient-sensing signaling pathways. (**A**) Protein expression of AKT and pAKT in adult males raised on diets with different yeast concentrations during development as indicated. (**B**) Quantifications of the densitometry of the Western blots of (**A**). (**C**) Protein expression of pS6K and S6K from males raised on diets with different yeast concentrations during development as indicated. (**D**) Quantifications of the densitometry of the Western blots in (**C**). Samples were collected at the time point of eclosion, mated for 48 h (day 0) and day 10. Flies on 1SY food mated for 48 h after eclosion. Then, male flies were transferred to 1SY or 3SY for 10 days. Data in (**B**,**D**) are shown as mean ± SEM. *p*-values were determined using an unpaired Student’s *t*-test. (**E**) Lifespan curve of adult male flies under low-yeast (0.2SY) and normal-yeast (1SY) diets during developmental stages. Chico heterozygous mutant flies (the F1 generation of chico-mutant flies crossed with *W^Dah^* flies) and *W^Dah^* control flies were used. (**F**) The lifespan curve of adult male flies under the same conditions as (**E**). AKT heterozygous mutant flies and *W^1118^* control flies were used. (**G**) The lifespan curve of adult male flies under the same conditions as (**E**). S6K heterozygous mutant flies and *W^1118^* control flies were used. (**H**) The lifespan curve of adult male flies under the same conditions as (**E**). Thor homozygous mutant flies and *W^1118^* control flies were used. Lifespan differences were assessed using the log-rank test. Lifespan data can be seen in the Tables S5–S7. * *p* < 0.05; ** *p* < 0.01; *ns*: not significant (*p* > 0.05).

We next explored the molecular mechanisms by which a low-yeast developmental diet extends the lifespan of male *Drosophila*. We hypothesized that male flies fed with 0.2SY LYDDS exhibit a longevity phenotype due to a lower response to nutritional signals when faced with nutrient stress. The key components of nutrient-sensing signaling pathway include the IIS and mTOR signaling pathways, which are also important in the developmental stage that controls growth [58,59]. We found that there was no significant difference in the protein expression of phosphorylated Akt kinase (pAKT) in flies immediately after eclosion between low-yeast (0.2SY) and normal-yeast (1SY) diets (Figure 4A,B). However, using a low developmental diet, pAKT levels were significantly reduced in flies on a 1SY diet that mated after 48 h or after 10 days of 1SY or 3SY (Figure 4A,B). The findings demonstrate that the IIS pathway of flies fed with 0.2SY LYDDS is suppressed in adulthood in response to an adult nutrient-replete diet. On the other hand, the expression levels of phosphorylated ribosomal protein S6 kinase (pS6K) were significantly decreased in flies immediately after eclosion fed with 0.2SY LYDDS diet compared to the 1SY and 3SY developmental diets (Figure 4C,D). When flies on 1SY adult food mated after 48 h, their pS6K levels still maintained a trend of decrease from 0.2SY LYDDS. However, at the day 10, the pS6K expression of flies did not showed become significantly different from those on adult diets of 1SY or 3SY among different larval diets (Figure 4C,D). Our findings illustrate that flies fed 0.2SY LYDDS had a lower mTOR signaling expression after eclosion and that this phenotype is gradually abolished by adult diets.

To determine whether the IIS or mTOR pathways mediate the lifespan extension of male flies due to the low-yeast diet during the developmental stages, we used heterozygous mutant flies of chico and AKT whose IIS pathway was inhibited and heterozygous mutant flies of S6K and homozygous viable Thor mutant [60] (homologous to mammal 4EBP) downstream of the mTOR signaling pathway to conduct lifespan validation under 0.2SY LYDDS. The results display that low yeast (0.2SY) during development could still prolong the lifespan of male flies of chico, AKT, S6K, and Thor mutants, although the extent of lifespan extension was reduced in AKT, S6K, and Thor mutant flies compared to the *W^Dah^* and *W^1118^* strains, respectively (Figure 4E–H and Tables S5 and S6). Among them, the lifespan-extending effect of a low-yeast developmental diet was almost abolished by the knocking down of S6K even if the lifespan of S6K heterozygous mutant flies fed 0.2SY LYDDS is still significantly longer (Figure 4G and Table S7). The above results suggest the lifespan-extending effect under nutritional programming is partially dependent on the IIS and mTOR signaling pathways.

### 3.5. dFOXO Activity Is Enhanced with the Low-Yeast Diet during Development

**Figure 5:**
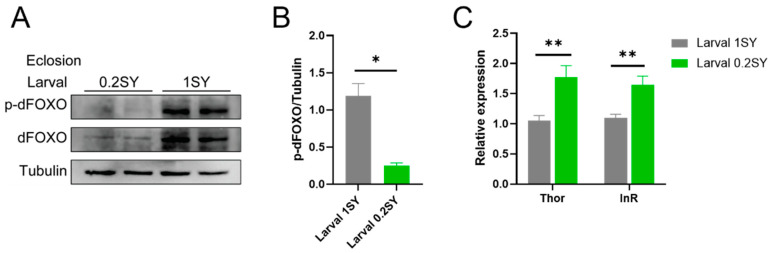
dFOXO activity is upregulated in response to a developmental low-yeast diet. (**A**) The protein expression of p-dFOXO and dFOXO from male flies raised on developmental low-yeast (0.2SY) and normal-yeast (1SY) diets. (**B**) Quantification of p-dFOXO and tubulin ratios for (**A**). (**C**) Transcriptional expression of dFOXO target genes InR and Thor. Data shown as mean ± SEM. *p*-values were determined by an unpaired Student’s *t*-test. * *p* < 0.05; ** *p* < 0.01.

It was previously reported that the transcription factor FOXO, downstream of insulin and insulin-like growth factor signaling, plays a key role in adapting metabolism to nutritional conditions and determining aging and longevity [61]. We speculated that dFOXO could be involved in the nutritional programming of a larval low-yeast diet. To confirm this role, we analyzed the protein expression of dFOXO in adult male flies. Interestingly, phosphorylated dFOXO (p-dFOXO, an inactive form of dFOXO) was significantly decreased in adult males under the low-yeast diet (0.2SY) compared to those on the normal-yeast larval food (1SY) during their development stages (Figure 5A,B). We further examined the transcriptional levels of insulin receptor (InR) and Thor, two known target genes of dFOXO [62,63]. The overall expression of InR and Thor were significantly upregulated in adulthood under 0.2SY LYDDS (Figure 5C). The above results confirm that dFOXO is activated in adult males fed with 0.2SY LYDDS.

### 3.6. dFOXO Is Required for Nutritional Programming for Lifespan Extension with a Low-Yeast Diet during Development

**Figure 6:**
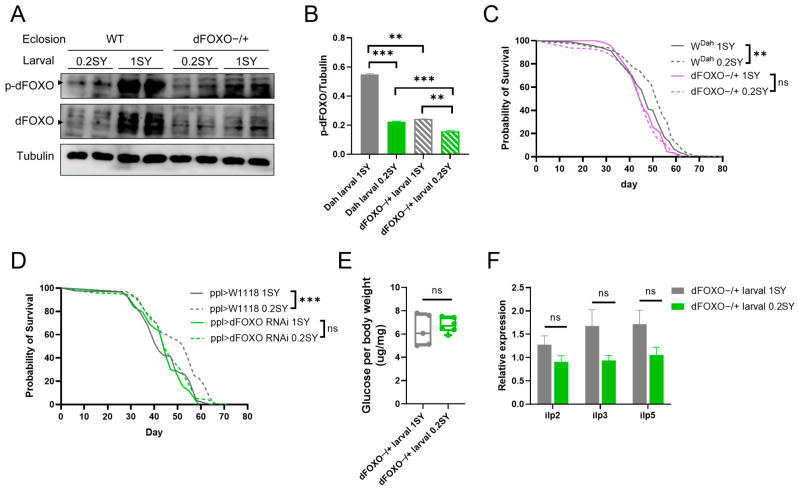
FOXO is required for lifespan extension effects on physiological phenotypes in response to a developmental low-yeast diet. (**A**) The protein expression of p-dFOXO and dFOXO from *W^Dah^* and dFOXO heterozygous mutant male flies after eclosion fed with low-yeast (0.2SY) and normal-yeast (1SY) diets during their developmental stages. (**B**) Quantification of p-dFOXO and tubulin ratios for (**A**). (**C**) The lifespan curve of dFOXO heterozygous mutant male flies and control flies raised on developmental low-yeast and normal-yeast diets. (**D**) The lifespan curve of dFOXO-knockdown male flies in the fat body fed with low-yeast and normal-yeast diets during developmental stages. dFOXO RNAi was expressed in the fat body via the fat-body-specific driver ppl-GAL4. (**E**) Glucose levels of dFOXO heterozygous mutant male flies raised on developmental low-yeast (0.2SY) and normal-yeast (1SY) diets. (**F**) The transcriptional level of insulin-like peptides from dFOXO heterozygous mutant male flies raised on developmental low-yeast and normal-yeast diets. Adult male samples of (**E**,**F**) were collected after flies on the 1SY diet mated for 48 h. Data in (**E**,**F**) shown as mean ± SEM. *p*-values were determined by an unpaired Student’s *t*-test. Lifespan differences were assessed using the log-rank test. Lifespan data can be seen in the Tables S8 and S9. ** *p* < 0.01; *** *p* < 0.001; *ns*: not significant (*p* > 0.05).

To further confirm whether dFOXO is involved in the lifespan-extending effects of 0.2SY LYDDS, we used the heterozygous dFOXO mutant (dFOXO−/+) for validation because homozygotes for dFOXO deletion (dFOXO−/−) under 0.2SY LYDDS were not viable as adults. The results show that p-dFOXO levels in both low-yeast (0.2SY) and normal-yeast (1SY) diets during development was significantly reduced in the dFOXO mutant compared to wild-type (*W^Dah^*) flies (Figure 6A,B). At the same time, p-dFOXO levels in mutant flies were significantly decreased when comparing those of flies fed with 0.2SY LYDDS and with a larval normal-yeast diet (Figure 6A,B). On the other hand, the difference in expression level of p-dFOXO in mutant flies between larval low-yeast and normal-yeast diets was low in comparison with wild-type flies fed with the same diets (Figure 6A,B). The lifespan-extending effect of 0.2SY LYDDS was fully abolished in dFOXO mutant male flies (Figure 6C and Table S8). Then, we performed the knockdown of dFOXO in the fat body, an adipose-like tissue in which dFOXO plays an important role, by using the fat-body-specific driver ppl-GAL4 and dFOXO RNAi flies. The results revealed that 0.2SY LYDDS does not prolong the lifespan in males with dFOXO knockdown in the fat body (Figure 6D and Table S9). Furthermore, the beneficial metabolic phenotypes of reduced glucose and reduced expression of insulin-like peptides are abolished in dFOXO mutant flies fed with 0.2SY LYDDS (Figure 6E,F). Taken together, these results indicate that dFOXO is required for the lifespan extension of the nutritional programming using 0.2SY LYDDS.

## 4. Discussion

We used *Drosophila* as a model organism to examine the mechanism by which nutritional programming impacts the adult lifespan and its underlying molecular mechanisms. Our results show that 0.2SY LYDDS can extend the lifespan and health span of male *Drosophila* under nutrient-replete conditions in adulthood. Simultaneously, male flies developed on a low-yeast diet caused an altered glucose metabolism, a lower overall glucose level, and reduced expression of the insulin-like peptides ilp3 and ilp5 during adulthood. We further demonstrated that the lifespan and health span of adult males fed with 0.2SY LYDDS were involved in nutrient-sensing signals, such as IIS and mTOR signaling. Importantly, we uncovered that a low-yeast diet during developmental stages stimulates the activity of dFOXO in male flies, which is required for lifespan extension. Collectively, we revealed dFOXO as a novel molecular mechanism for the nutritional programming of the lifespan in male *Drosophila*. FOXO could serve as a potential therapeutic target for the programming of lifelong health.

In *Drosophila*, it is well-documented that a larval low-yeast diet can alter various life-history traits—for example, lower body weight [16] and lower fecundity [52,53]. Our study confirmed that low- or high-yeast-concentration diets during development reduced body weight and egg production in adulthood. The lower fecundity fitness may be associated with a reduced number of ovarioles in flies developing on a low-yeast diet [14]. In addition, we also showed that flies developed on low-yeast diets stored more lipids and displayed more resistance to starvation in adulthood, which is consistent with previous reports [53,64]. Furthermore, we found that developmental low-yeast diets can delay climbing ability decline with age and improve adult glucose metabolism. These improvements in physiological functions are similar with those in DR adults [3,54]. It demonstrates that flies developed on low-yeast diets had improved physiological functions in adulthood. Previous studies in mice showed that early access to lower nutrition via increasing litter size or the maternal feeding of a low-protein diet during lactation significantly increased the lifespan of the offspring [65]. It has also been observed that *Drosophila* developing on poor larval food only increased adult virgin lifespan, not that of mated flies [15]. However, another report showed that a low-yeast diet only increased the lifespan of male flies, not that of female flies [66]. It was reported that the diluted nutrition of a larval diet can extend the lifespan of male and female flies [16,67]. Our results further establish that a low-yeast diet during development extends the health span and lifespan of male flies under nutrient-replete conditions in adulthood.

Our results also demonstrate that the activities of the IIS and mTOR signaling pathways were suppressed for male flies developed on a low-yeast diet after eclosion. Deregulated nutrient sensing is one of the hallmarks of aging [68]. Reducing the activity of the IIS and mTOR signaling pathways, two important nutrient-sensing signaling pathways, can promote healthy aging [3]. It is well-established that inhibiting the IIS and mTOR pathways can extend lifespan in numerous species, from invertebrates to mammals [69]. At the same time, IIS and mTOR are considered to mediate the lifespan extension effect of DR [33]. In our study, through reducing the activity of the key AKT components of IIS, S6K, and Thor of the mTOR signaling pathways, the lifespan extending effect of the low-yeast diet during development was partially abolished. This suggests that a low-yeast diet during development and DR share partially overlapping mechanisms, because 0.2SY LYDDS does not further extend the maximum median lifespan achieved in adulthood under the optimal adult diet of 0.2SY. There are limitations to our study. The lifespan of fruit flies is affected by a variety of complex factors. Among them, the genetic background may interfere with the comparison of lifespans between different fruit fly strains. Therefore, it is ideal that mutants or transgenes are backcrossed into a well-characterized, wild-type inbred strain for at least ten generations to ensure the homogeneity of the genetic background [70,71,72,73]. The insufficient number of backcross generations in some of our experiments makes it tricky to evaluate the precise contribution of the nutrient-sensing pathways to the longevity induced by 0.2SY LYDDS.

FOXO acts as a central integrative hub in response to cellular stimuli to regulate multiple cellular processes, including metabolism, protein homeostasis, intracellular signaling, apoptosis, and cell cycle arrest [39]. Although FOXO3 has been confirmed as a mediator of the life extension effect of diet restriction in mice [41], dFOXO is not required for lifespan extension by DR in *Drosophila* [74]. However, dFOXO is involved in mediating normal responses to DR, possibly by altering the expression of its target genes [74]. Our study revealed that dFOXO is required for both lifespan extension and improved glucose metabolism phenotype on a low-yeast diet during development, suggesting dFOXO as a key regulator involved in the nutritional programming of health span and lifespan. It was reported that FOXO mediates the long-term detrimental effects of a high-sugar diet in early adulthood on lifespan and that FOXO marks dietary history by programming downstream gene transcription [7]. This suggests a potential regulatory role of FOXO in nutritional memory. Nutritional programming effects may be expressed through specific tissues. Studies in aged mice suggest that the transcriptional memory of early adult nutrition maintained in adipose tissues (especially in white adipose tissue) counteracts the benefits of DR in aged mice [5]. After the knockdown of dFOXO in the fat body, we found that the lifespan-extending effects of a low-yeast diet during development were completely abolished. This suggests that the fat body may be an important target tissue for developmental nutritional programming. Taken together, our results reveal molecular evidence for FOXO’s involvement in the nutritional programming of the lifespan in male *Drosophila*. The mechanism through which FOXO programs the transcription of downstream genes and its contribution to the nutritional programming of health and lifespan need to be further studied.

## Figures and Tables

**Table 1:** The list of the primers.

Primer Name	Primer Sequence
Nlaz F:	GGACAACCCTCGAATGTAACT
Nlaz R:	GACGGCGTATGACTCGTAATC
dilp2 F:	CCTGCAGTTTGTCCAGGAGT
dilp2 R:	AGCCAGGGAATTGAGTACACC
dilp3 F:	GTATGGCTTCAACGCAATGAC
dilp3 R:	GAGCATCTGAACCCAACTATCAC
dilp5 F:	CGTGATCCCAGTTCTCCTGT
dilp5 R:	ACCCTCAGCATGTCCATCAA
Thor F:	CCAGGAAGGTTGTCATCTCG
Thor R:	TGAAAGCCCGCTCGTAGATA
InR F:	GGTGCTGGCATCATAGGTCT
InR R:	CCTGCCTCTGAGTGATAGAAGG
RP49 F:	GCCCAAGGGTATCGACAACA
RP49 R:	CTTGCGCTTCTTGGAGGAGA

## Data Availability

Not applicable.

## Supplementary Materials

The following supporting information can be downloaded at: https://www.mdpi.com/article/10.3390/nu15081840/s1. Figure S1: Body weight and lifespan curves. Figure S2: Developmental low yeast promotes the health of male flies. Tables S1–S9: Fly lifespan statistical data.
