# Supplementary material for: Nutritional Programming of the Lifespan of Male Drosophila by Activating FOXO on Larval Low-Nutrient Diet
Source: Nutrients. 2023 Apr 11;15(8):1840. doi: 10.3390/nu15081840 (PMC10142539; doi:10.3390/nu15081840)
Supplement: Supplementary file 1 [file nutrients-15-01840-s001.zip › nutrients-2241971-supplementary.pdf]

Nutritional programming of the lifespan of male Drosophila by activating FOXO on larval low-nutrient diet

Yue Gao [1], Xingyi Cheng [1], Yao Tian [1], Zhixiao Yuan [1], Xiaolan Fan [1,2], Deying Yang [1,2], and Mingyao Yang [1,2,*]

Supplementary Figures

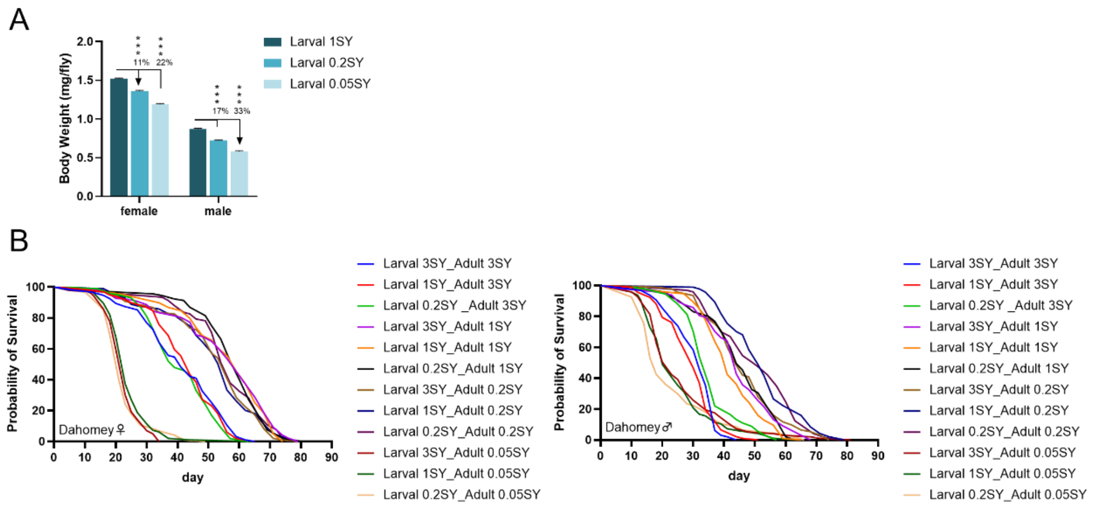

Figure S1. Body weight and lifespan curves. (A) Body weight of males after mating for 48 hours (day0) under different developmental diet of 0.05SY, 0.2SY, 1SY. (B) Female (left) and male (right) lifespan curve of Figure1D-E. All results shown are of *Dahomey* flies. Body weight in (A) showed as mean ±SEM. p values were determined by unpaired Student's t test for body weight data. Lifespan differences were assessed using the log-rank test. Lifespan data can be seen at the Tables S1,2. *p < 0.05; **p < 0.01; ***p < 0.001; ****p < 0.0001.

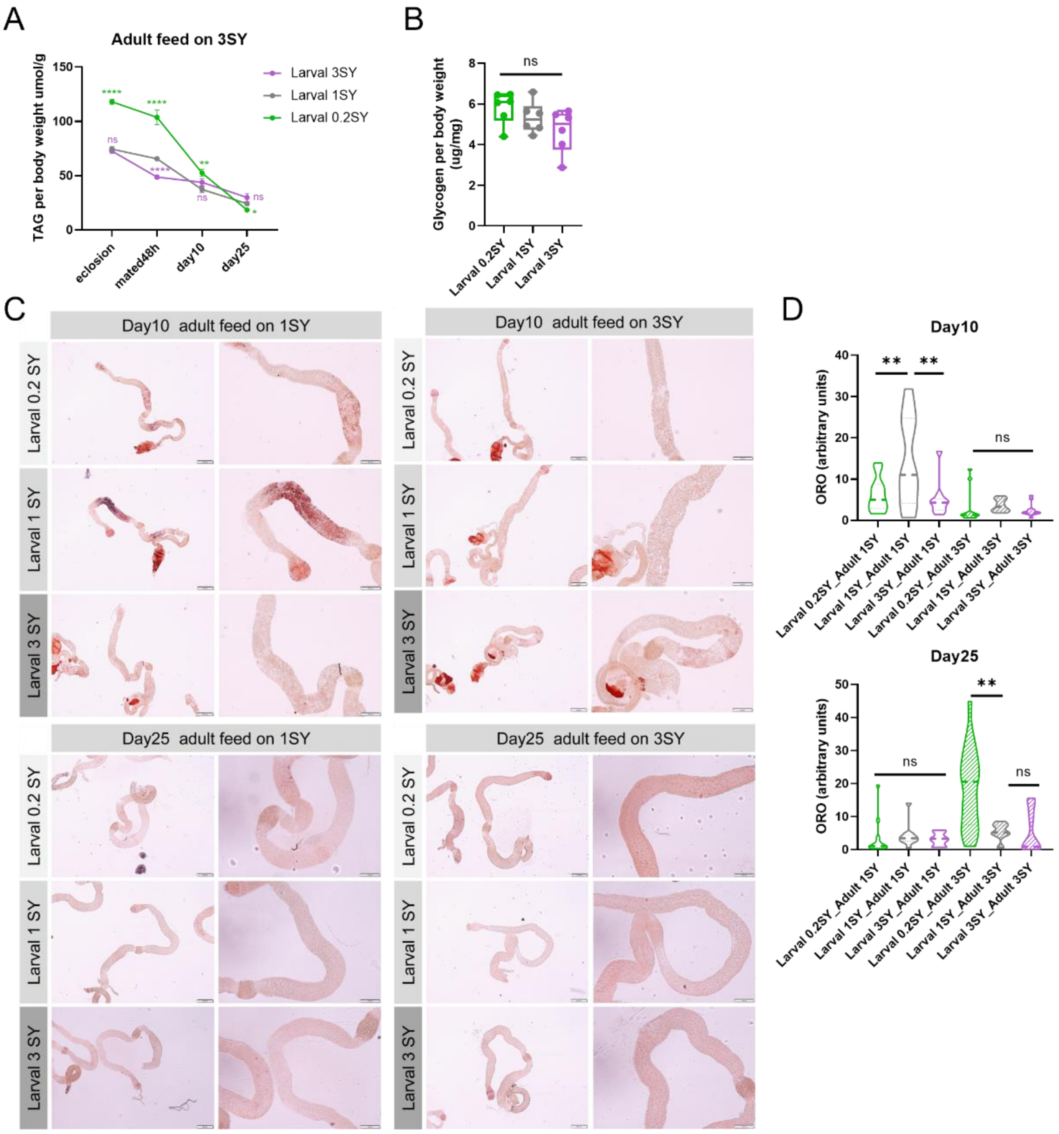

Figure S2. Developmental low yeast diet promotes health of male flies. (A) Total TAG content per body weight of males in different developmental yeast concentrations under 3SY diet during adulthood. Samples were collected at time point of eclosion, mated for 48 hours, day 10, day 25. (B) Glycogen levels per body weight were not significantly different in males after mating for 48 hours under different yeast concentration diets in development. (C) Oil Red O staining gut lipid of day 10 and day 25 male flies raised on 0.2SY, 1SY or 3SY diet during developmental stages. Adult diets were 1 SY and 3SY respectively. (D) Quantification of the ORO signal in the anterior midgut. At least 7 guts were quantified for each condition. All results shown are of WT *Dahomey* male flies. Adult diets are 1SY or 3SY food as indicated. Data in (A, B) showed as mean ±SEM. Each point of (B) represents 1 vial of 10 flies. p values were determined by unpaired Student's t test (for A, B) or one-way ANOVA followed by Sidak multiple comparisons (for quantification data of ORO signal). $^{*}p < 0.05$; $^{**}p < 0.01$; $^{***}p < 0.001$. $^{****}p < 0.0001$.

Supplementary Tables

Lifespan statistical data

Table S1

Lifespan data related to Figure 1D and Figure S1B

		Female											
		Larval 3SY _Adult 3SY	Larval 1SY _Adult 3SY	Larval 0.2SY _Adult 3SY	Larval 3SY _Adult 1SY	Larval 1SY _Adult 1SY	Larval 0.2SY _Adult 1SY	Larval 3SY _Adult 0.2SY	Larval 1SY _Adult 0.2SY	Larval 0.2SY _Adult 0.2SY	Larval 3SY _Adult 0.05SY	Larval 1SY _Adult 0.05SY	Larval 0.2SY _Adult 0.05SY
Total deaths		95	99	100	97	99	92	98	99	92	99	96	95
Median survival (d)		41	44	39	59	59	60	56	56	57	23	23	21
Log-rank (Mantel-Cox) test /Gehan-Breslow-Wilcoxon test	(vs Larval 1SY _Adult 3SY)	0.9747 /0.4642		0.2616 /0.2038									
	(vs Larval 1SY _Adult 1SY)				0.5902 /0.9126		0.9560 /0.5974						
	(vs Larval 1SY _Adult 0.2SY)							0.9587 /0.7389		0.1647 /0.0647			
	(vs Larval 1SY _Adult 0.05SY)										0.0765 /0.1473		0.0840 /0.0144

Table S2

Lifespan data related to Figure 1E and Figure S1B

		Male											
		Larval 3SY _Adult 3SY	Larval 1SY _Adult 3SY	Larval 0.2SY _Adult 3SY	Larval 3SY _Adult 1SY	Larval 1SY _Adult 1SY	Larval 0.2SY _Adult 1SY	Larval 3SY _Adult 0.2SY	Larval 1SY _Adult 0.2SY	Larval 0.2SY _Adult 0.2SY	Larval 3SY _Adult 0.05SY	Larval 1SY _Adult 0.05SY	Larval 0.2SY _Adult 0.05SY
Total deaths		96	92	91	98	94	95	93	94	97	97	96	94
Median survival (d)		32	30	35	44	41	44	44	51	51	23	20	18
Log-rank (Mantel-Cox) test /Gehan-Breslow-Wilcoxon test	(vs Larval 1SY _Adult 3SY)	0.387 /0.1459		<0.0001 /<0.0001									
	(vs Larval 1SY _Adult 1SY)				0.0409 /0.1371		0.0083 /0.0196						
	(vs Larval 1SY _Adult 0.2SY)							0.004 /0.0009		0.7903 /0.4780			
	(vs Larval 1SY _Adult 0.05SY)										0.3632 /0.5967		0.5634 /0.0370

Table S3

Lifespan data related to Figure 1F

		Dahomey ♂					
		Larval 3SY _Adult 1SY	Larval 1SY _Adult 1SY	Larval 0.2SY _Adult 1SY	Larval 3SY _Adult 3SY	Larval 1SY _Adult 3SY	Larval 0.2SY _Adult 3SY
Total deaths		98	100	96	95	92	95
Median survival (d)		42	42	51	29	29	33
Log-rank (Mantel-Cox) test /Gehan-Breslow-Wilcoxon test	(vs Larval 1SY _Adult 1SY)	0.9854 /0.7121		<0.0001 /<0.0001			
	(vs Larval 1SY _Adult 3SY)				0.8722 /0.7546		0.0002 /0.0003

Table S4

Lifespan data related to Figure 1H

	Dahomey ♂		
	Larval 3SY _Adult Yaa	Larval 1SY _Adult Yaa	Larval 0.2SY _Adult Yaa
Total deaths	97	96	96
Median survival (d)	43	41.5	46
Log-rank (Mantel-Cox) test (vs Larval 1SY _Adult Yaa)	0.6128		<0.0001
Gehan-Breslow-Wilcoxon test (vs Larval 1SY _Adult Yaa)	0.2934		<0.0001

Table S5

Lifespan data related to Figure 4E

		Male			
		W^{Dah} 1SY	W^{Dah} 0.2SY	chico −/+ 1SY	chico −/+ 0.2SY
Total deaths		99	96	98	95
Median survival (d)		45	48	53	59
Log-rank (Mantel-Cox) test /Gehan-Breslow-Wilcoxon test	W^{Dah} 1SY		0.0016 /0.0024	<0.0001 /<0.0001	
	W^{Dah} 0.2SY				<0.0001 /<0.0001
	chico −/+ 1SY				0.0001 /0.0004
	chico −/+ 0.2SY				

Table S6

Lifespan data related to Figure 4F and 4H

		Male					
		W1118 1SY	W1118 0.2SY	AKT−/+ 1SY	AKT−/+ 0.2SY	Thor−/− 1SY	Thor−/− 0.2SY
Total deaths		99	97	99	69	98	93
Median survival (d)		31	41	33	37	34	38
Log-rank (Mantel-Cox) test /Gehan-Breslow-Wilcoxon test	W1118 1SY		<0.0001 /<0.0001	0.0564 /<0.0001		0.0003 /0.0001	
	W1118 0.2SY				<0.0001 /<0.0001		<0.0001 /0.0036
	AKT−/+ 1SY				0.0091 /0.0012		
	Thor−/− 1SY						0.0173 /0.0133

Table S7

Lifespan data related to Figure 1G and Figure 4G

		Male			
		W1118 1SY	W1118 0.2SY	S6K−/+ 1SY	S6K−/+ 0.2SY
Total deaths		101	96	98	89
Median survival (d)		39.5	56.5	47.5	50
Log-rank (Mantel-Cox) test /Gehan-Breslow-Wilcoxon test	W1118 1SY		<0.0001 /<0.0001	0.2669 /0.0003	
	W1118 0.2SY				<0.0001 /<0.0001
	S6K−/+ 1SY				0.0390 /0.2187
	S6K−/+ 0.2SY				

Table S8

Lifespan data related to Figure 6C

		Male			
		W^{Dah} 1SY	W^{Dah} 0.2SY	dFOXO−/+ 1SY	dFOXO−/+ 0.2SY
Total deaths		90	100	90	98
Median survival (d)		48	54	46	45
Log-rank (Mantel-Cox) test /Gehan-Breslow-Wilcoxon test	W^{Dah} 1SY		0.0087 /0.0118	0.2003 /0.4634	
	W^{Dah} 0.2SY				<0.0001 /<0.0001
	dFOXO−/+ 1SY				0.6902 /0.4578
	dFOXO−/+ 0.2SY				

Table S9

Lifespan data related to Figure 6D

		Male			
		ppl>W1118 1SY	ppl>W1118 0.2SY	ppl>dFOXO RNAi 1SY	ppl>dFOXO RNAi 0.2SY
Total deaths		85	96	87	96
Median survival (d)		42	54	45	45
Log-rank (Mantel-Cox) test /Gehan-Breslow-Wilcoxon test	ppl>W1118 1SY		0.0002 /0.0019	0.9727 /0.6769	
	ppl>W1118 0.2SY				0.0188 /0.0474
	ppl>dFOXO RNAi 1SY				0.3682 /0.4903
	ppl>dFOXO RNAi 0.2SY				
